# Evaluation of an AI-Supported Nutrition Application (WiseFood) in a Living Lab Context: Protocol for a User Needs Assessment, Co-Design, and Feasibility Testing

**DOI:** 10.2196/88810

**Published:** 2026-04-27

**Authors:** Niamh M Walsh, Pauline Dunne, Cathal O'Hara, Saša Štraus, Tamara Kozic, Emese Antal, Vanda Pózner, András Vig, Dávid Szakos, Stylianos Kolidakis, Dimitrios Skoutas, Angela C Flynn, Claire M Timon

**Affiliations:** 1 School of Population Health RCSI University of Medicine and Health Sciences Dublin 2 Ireland; 2 Innovation Technology Cluster Murska Sobota (ITC) Murska Sobota Slovenia; 3 ESSRG Nonprofit Kft Budapest Hungary; 4 Wasteless Foundation Budapest Hungary; 5 Department of Applied Food Sciences Institute of Food Chain Science University of Veterinary Medicine Budapest Budapest Hungary; 6 Athena Research and Innovation Center In Information Communication & Knowledge Technologies Marousi Greece

**Keywords:** artificial intelligence, AI, co-design, living labs, nutrition, sustainability

## Abstract

**Background:**

Unhealthy and unsustainable diets remain a major global challenge, contributing significantly to poor health outcomes, environmental degradation, and social inequalities. Despite growing awareness, individuals face persistent barriers to adopting sustainable dietary practices, including cost, availability, cultural norms, and low food literacy. While digital tools and artificial intelligence (AI) offer promising avenues to support dietary behavior change, few interventions target the household as a unit of change. The WiseFood project addresses this gap by developing AI-supported apps to promote healthier and more sustainable food choices at the household level through co-designed interventions in multisite Living Labs (LLs) across Europe.

**Objective:**

The WiseFood project aims to co-design, develop, and test the feasibility of an AI-supported digital platform to promote sustainable, healthy diets at the household level. This protocol outlines the recruitment of stakeholders, the user needs and requirements phase, the co-design phase, and the feasibility study phase.

**Methods:**

The WiseFood project follows a 4-phase design across 3 LL sites in Ireland, Hungary, and Slovenia. Phase 1 involves the recruitment of diverse stakeholders, including households and experts for co-design activities. In Phase 2, user needs and requirements are assessed through household surveys and expert focus groups exploring AI in nutrition. Phase 3 consists of co-design workshops and iterative feedback loops to refine the WiseFood digital tools. Phase 4 is an 8-week feasibility study involving 300 households (n=100 per site), evaluating usability, acceptability, and outcomes related to nutrition knowledge, environmental awareness, and dietary behaviors. Data will be collected at baseline and postintervention using validated surveys.

**Results:**

The 3-year project (January 1, 2025-December 31, 2027) follows a 4‑phase structure to develop, refine, and test a user‑focused app across LL sites in Ireland, Hungary, and Slovenia. Phase 1 was completed in May 2025, while Phase 2 ran from June to July 2025. Phase 3, which commenced in September 2025, is expected to continue until June 2026. Phase 4 will commence in July 2026, and will run through to November 2027. The findings from the co-design and feasibility phases will be published separately and will include insights into usability, acceptability, and changes in nutrition knowledge, environmental awareness, and dietary behaviors. These results will inform further refinement of the WiseFood platform and guide future implementation and evaluation efforts.

**Conclusions:**

The WiseFood project adopts an evidence-based approach to develop AI-supported digital apps that encourage informed, healthy, and sustainable food practices in the home. By considering the differing needs of household members, WiseFood advances applied approaches that deliver targeted support in everyday household contexts.

**International Registered Report Identifier (IRRID):**

DERR1-10.2196/88810

## Introduction

### Background

Diet-related noncommunicable diseases, strongly associated with diets high in animal-based and processed foods, constitute the leading cause of global mortality. These dietary patterns also contribute substantially to environmental burdens [1,2]. The European Union’s Farm-to-Fork strategy aims to accelerate the transition to sustainable, healthy, and inclusive food systems. Its goals include halving per capita food waste (FW) at retail and consumer levels by 2030 and promoting a shift toward more plant-based diets to reduce diet-related diseases and environmental impacts [1]. However, we are still far from achieving this goal, both at the European and global level, with persistent inequalities in access to nutritious food [1]. Globally, around 800 million people go hungry and are underweight or malnourished [3]. Yet, at the same time, 2 billion people are overweight or obese [3]. In the European Union, 1 in 4 people are at risk of poverty or social exclusion, and only 9.1% can only afford a quality meal every second day, yet 33% of our total food is wasted [3,4].

Sustainable healthy diets are defined as dietary patterns that promote health and well-being, exert a low environmental impact, and are economically fair, affordable, and culturally acceptable [4]. These diets are characterized by a high proportion of seasonal and locally produced plant-based foods, with a lower proportion of animal-sourced foods and highly processed foods [5]. Despite increasing recognition of the need for healthy and sustainable diets, current food systems continue to promote dietary patterns that are environmentally damaging and nutritionally inadequate; contributing up to 30% of global greenhouse gas emissions and leading to widespread overconsumption of processed foods and underconsumption of diverse, nutrient-rich plant-based foods [4-6]. Household food consumption is often not a sustainable practice, with more than one-fourth of food produced globally wasted; for instance, in Hungary, households generate just under 60 kg of FW per person annually, of which 21.5 kg could be avoided through more conscious consumer behavior, in Slovenia, FW is increasing yearly, with 52% of all waste generated at the household level, and in 2023, Irish households discarded approximately 120 kg of FW per household [7-9].

Numerous studies have identified consistent barriers to adopting sustainable healthy diets, such as concerns about taste, cost, availability, time constraints, ingrained habits, low food literacy, distrust of information sources, and competing priorities like health, convenience, and finances [10-12]. For example, in a UK qualitative observational study involving 21 participants, there was reluctance to reduce meat consumption due to perceived loss of enjoyment, alongside perceived challenges to eating sustainably such as higher costs, limited availability, and the effort required for sourcing and preparation [12]. While participants were open to making their diets more sustainable, they expressed uncertainty about how to do so, highlighting the need for increased awareness and clearer guidance around sustainable healthy diets [12].

In the literature to date, a number of approaches have been used to increase the consumption of sustainable healthy diets. For example, Sullivan [13] designed an intervention targeting healthy adults in Ireland to encourage adherence to a healthy diet with a low environmental impact. In this study, the provision of personalized sustainable dietary advice led to healthier diets and lower diet-related greenhouse gas emissions [14]. However, the study cohort was predominantly White, urban-based, and highly educated. Similarly, Monroe et al [15] implemented an intervention among US students focused on the promotion of green eating (GE) behaviors, including FW reduction and the acquisition of local foods from farmers’ markets. GE is defined as eating locally grown foods, limiting processed foods, eating meatless meals at least once per week, choosing organic foods when possible, and only taking what you plan to eat. This intervention significantly improved GE behaviors in the short term, with students showing more environmentally conscious attitudes and actions. However, it did not address overall dietary patterns, focusing mainly on red meat consumption and excluding broader measures of dietary quality [15]. While both O’Sullivan and Monroe’s [13,15] interventions offer valuable strategies for promoting sustainable, healthy diets, their implementation in structured settings and a more focused target population may influence their applicability to broader, real-world contexts. At the European Union level, the PLAN’EAT project aims to better understand how environmental, social, cultural, and individual factors affect people’s food choices and will co-design interventions to effectively reverse trends in suboptimal dietary behavior [16].

The general public is increasingly relying on online sources of nutrition information despite concerns regarding the poor quality of much of that information [17-19]. Yet, there has been limited focus to date on the potential of digital and AI tools to support sustainable healthy diets. Fresán et al [20] piloted a smartphone-based intervention to promote sustainable diets among adults in Barcelona, showing improvements in dietary behaviors and awareness of food-related environmental and social factors. While promising, this study did not incorporate AI-driven personalization and was limited by a relatively homogenous sample, highlighting the need for future research in more diverse populations and settings.

While previous interventions have targeted individual behavior change, few have addressed the collective nature of household food decisions or leveraged AI to personalize dietary recommendations. Targeting households aligns with the interconnected nature of food choices within familial settings and offers a strategic approach to promoting healthier and more sustainable lifestyles at a broader societal level [21]. There appears to be a gap in the literature focusing on the development of technological solutions to support healthy and sustainable dietary choices at a family household level. Especially in the case of families, focusing on households as the target group has the potential to increase parents’ engagement and facilitate behavior changes in children [22,23]. Recent evidence suggests a digital approach at a household level is promising, with one paper showing that a family web-based nutrition intervention led to short-term improvements in dairy product intake and nutrient consumption among children aged 8-16 years [22]. These findings highlight the potential value in adopting a household-level approach, particularly through the use of digital tools, as a means of enhancing parental involvement and supporting healthier food choices.

The Horizon Europe WiseFood project [24] addresses the pressing need for healthier and more sustainable dietary habits, in line with global public health and environmental goals. By leveraging artificial intelligence (AI), WiseFood aims to empower households to make informed food choices through the creation of three applications: FoodScholar, RecipeWrangler, and FoodChat. These applications will be co-designed and evaluated within Living Labs (LLs). Unlike many digital nutrition interventions that focus on individual behavior change, provide static or generic advice, or rely on highly controlled research settings with limited real-world applicability, WiseFood aims to address these limitations by adopting a LL approach. As defined by the European Network of Living Labs, LLs are collaborative environments where relevant actors work together to develop, test, and validate new products or services in real-life settings [25]. Within this framework, the WiseFood applications will be co-designed and evaluated, ensuring that they reflect everyday household contexts and user needs. The WiseFood project uses this framework to bring together the views of citizens with scientific and technological knowledge, ensuring that the resulting digital tools are not only technically robust but also socially relevant, context-sensitive, and responsive to everyday household realities. This transdisciplinary, cross-cultural co-design process allows WiseFood to capture diverse dietary practices, affordability constraints, and sustainability perceptions, creating scalable, locally sensitive tools that are more likely to be accepted, adopted, and sustained beyond the project duration.

WiseFood contributes several key innovations that address limitations in other interventions, including targeting households as the unit of change, using AI-driven personalization for adaptive, culturally relevant recommendations, and applying a multicountry LL approach to co-design tools in real-life contexts. Together, these elements distinguish WiseFood from existing digital or behavioral nutrition interventions and directly address gaps in the sustainable healthy diets and digital health literature.

### Objective

**Figure 1:**
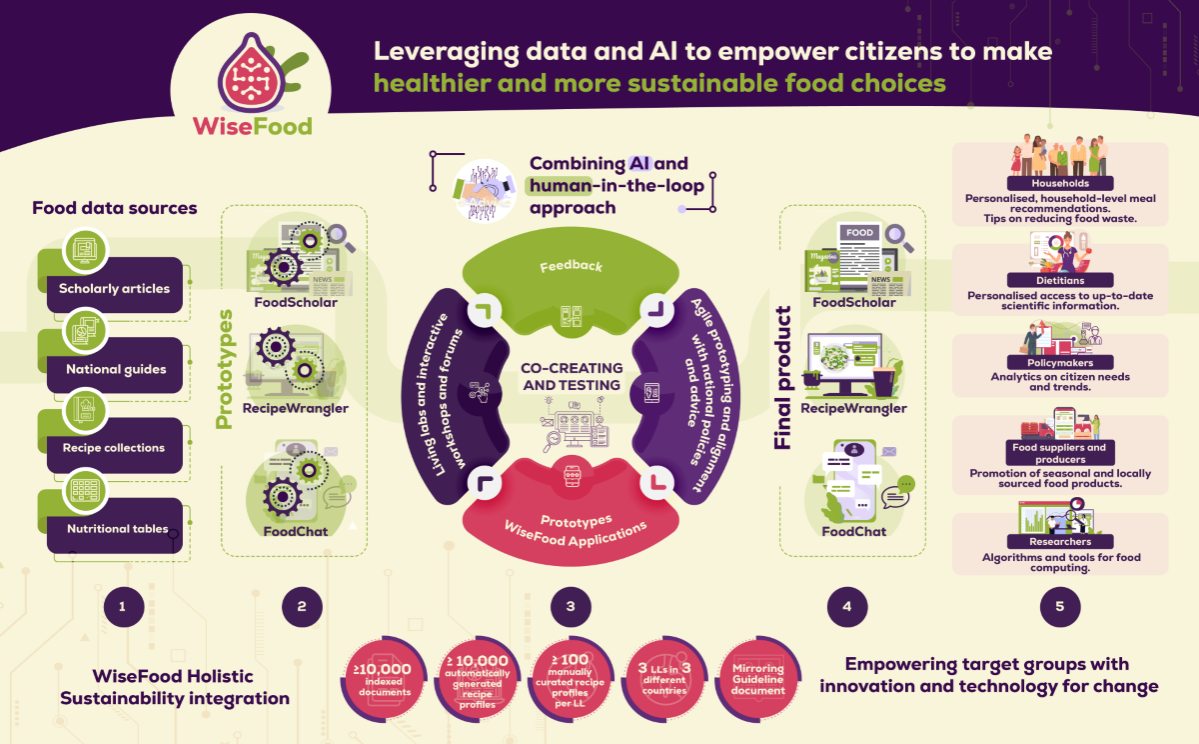
WiseFood concept overview. AI: artificial intelligence; LL: Living Lab.

The primary aim of the WiseFood project is to co-design, develop, and assess the feasibility of an AI-supported digital platform that promotes sustainable healthy diets at the household level. The secondary aim is to explore the potential effectiveness of the platform in informing and influencing household dietary behaviors, thereby generating evidence to support the design of future large-scale interventions focused on sustainability and health. An overview of the WiseFood approach is illustrated in Figure 1.

To achieve these aims, the project will:

1. Conduct co-design activities using the Bird et al [26] generative co-design framework involving stakeholders and end users.
2. Collaborate with technology partners to develop the digital platform and its supporting infrastructure.
3. Implement a multicenter, 8-week single-arm cohort feasibility study involving 300 households across three LL sites in Ireland, Hungary, and Slovenia.
4. Assess changes in household food knowledge and behavior in terms of nutrition and sustainability.

This protocol outlines the User Needs and Requirements (UNR) phase, co-design of the WiseFood applications, and the subsequent feasibility study, including information on study design, recruitment, data collection, and analysis.

## Methods

### Overview

**Figure 2:**
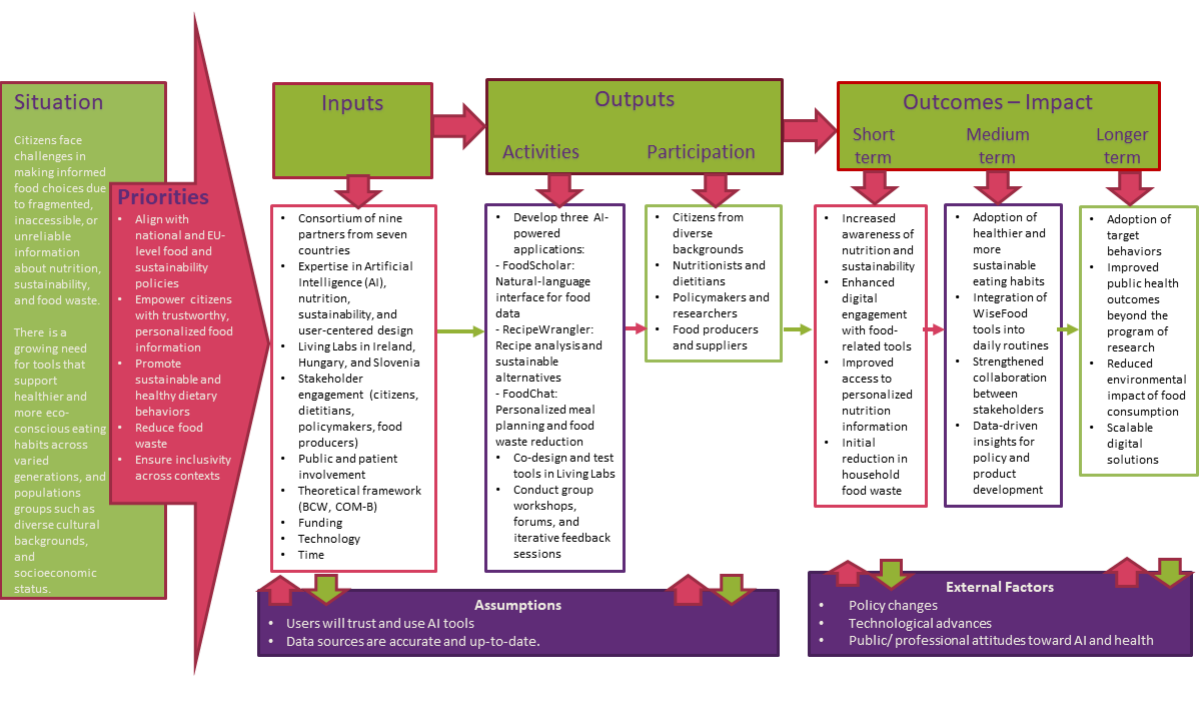
Logic model for the WiseFood project. AI: artificial intelligence; BCW: Behavior Change Wheel.

The WiseFood project will use a multisite LL approach to co-design and test its digital applications in real-world settings across Ireland (Royal College of Surgeons in Ireland; RCSI), Hungary (Environmental Social Science Research Group; ESSRG/Wasteless Foundation), and Slovenia (Green Point). This design allows for cross-cultural comparison, ensuring that the resulting tools are adaptable to diverse European food environments and user needs. Each LL site will conduct stakeholder engagement, co-design workshops, and feasibility testing tailored to their national context. RCSI will oversee coordination, methodological guidance, and synthesis of findings to identify common themes and country-specific insights. This collaborative structure supports the development of a culturally responsive, evidence-based platform adaptable to diverse European food environments. A WiseFood project-specific logic model was developed to illustrate the relationships between inputs, activities, outputs, and intended outcomes, and to guide implementation and evaluation across sites (Figure 2). It incorporates key inputs, such as a multicountry consortium with expertise in AI, nutrition, and sustainability, alongside stakeholder engagement. Activities focus on developing and testing 3 AI-powered nutrition tools with participation from citizens, health professionals, policymakers, and food producers. Expected outcomes range from improvements in nutrition and sustainability awareness and reduced household FW (short term) to adoption of sustainable eating habits (medium term), with potential for long-term public health and environmental benefits.

WiseFood applies the Behavior Change Wheel as its primary behavior change framework [27]. Using the COM-B system, the project targets capability through nutrition education and recipe transformation (FoodScholar and RecipeWrangler), opportunity through personalized meal planning (FoodChat), and motivation through conversational reinforcement [27]. Building on this, the Behavior Change Taxonomy provides a detailed set of techniques that translate these intervention functions into concrete, actionable strategies [28]. Behavior Change Taxonomies applied include information provision, behavioral substitution, self-monitoring, and positive reinforcement. This approach enables households to adopt healthier and more sustainable food practices through accessible, user-centered conversational AI tools.

### Ethical Considerations

Ethical oversight is led by the project coordinator and an appointed external ethics advisor, who will provide guidelines for ensuring that all partners adhere to the WiseFood ethical framework. All activities will be conducted in full accordance with the General Data Protection Regulation, as well as relevant national legislation, the Data Governance Act and AI Act. In order to maintain participant privacy, data sharing agreements were established between all project partners. Ethical approval for the study was granted by the RCSI Research Ethics Committee (REC202504022) in Ireland, the Research Ethics Committee of ITC Murska Sobota in Slovenia (EC-ITC-2025-01), and the Research and Innovation Committee of the University of Veterinary Medicine Budapest in Hungary (2025/08/27/1). A Data Protection Impact Assessment, conducted by the Royal College of Surgeons in Ireland (RCSI), further ensured compliance with data protection requirements and the safeguarding of trial participants’ data. Informed consent will be obtained from all participants prior to their involvement. Participants will receive detailed information about the WiseFood project, including its purpose, procedures, potential risks, and benefits. Informed consent will be obtained in accordance with institutional and ethical guidelines. Participation is voluntary, and individuals can withdraw at any time. To support participation, all contributors will receive a voucher incentive (eg, US $20 shopping voucher) upon completion of the survey or focus group and upon completion of Phase 3.

### Recruitment

Participants will be recruited for WiseFood via advertisements and posts on social media, engagement with community groups, and using researchers’ existing networks. Specific efforts will be made to reach underrepresented and vulnerable groups, including single-parent households and individuals from lower socioeconomic backgrounds (via community centers), older adults and rural populations (through age-action networks), professional groups, such as nutritionists, dietitians, policymakers, health care professionals, and food industry stakeholders (via direct contact, professional networks, and sector-specific events).

### Study Design

The study design is split into 4 distinct phases as outlined in Table 1.

**Table 1:** Overview of the 4 WiseFood project phases and associated activities.

Phase	Title	Main objectives and activities	Outputs
1	Establishment of Stakeholder panel	Recruit ~50 diverse stakeholders per LL^a^ to participate in the co-design process, comprising: ~ 40 diverse households. ~10 expert stakeholders (nutritionists, dietitians, policymakers, etc).	Active stakeholder panels at each LL site
2	User needs and requirements	Building on Phase 1, recruit panel members to participate in the user needs assessment. Household participants to complete an online survey on AI^b^ and food choices. Expert stakeholders to partake in focus groups to explore the feasibility, acceptability, and equity of AI in nutrition. Synthesize findings to refine WiseFood prototypes.	Comprehensive set of user needs and system requirements
3	Co-design workshops and iterative feedback loops	Conduct structured co-design workshops with stakeholder panel members to generate ideas and features for WiseFood using persona- and scenario-based approaches. Conduct iterative design cycles with technical partners and end users, resulting in a final prototype. Refinement of WiseFood prototypes based on workshops.	User-validated prototype of WiseFood applications
4	Feasibility study	Implement an 8-week single-arm cohort study to assess feasibility and acceptability of WiseFood prototypes. Recruit 300 households across 3 LL sites, incorporating diverse socioeconomic and cultural groups. Recruitment conducted separately from stakeholder panel to avoid bias. Participants complete informed consent and baseline measures. Households receive online training and access WiseFood via a dedicated website. Participants encouraged to use the tool regularly and document their experiences. Support provided throughout the study. Households repeat baseline measures and complete evaluation surveys after 8 weeks.	Evidence of feasibility, usability, and acceptability of WiseFood applications

^a^LL: Living Lab.

^b^AI: artificial intelligence.

### Phase 1 and Phase 2: Establishment of Stakeholder Panel and User Needs and Requirements

#### Study Overview

These phases of the WiseFood project will involve the establishment of stakeholder panels and the assessment of user needs and requirements across 3 LL sites: Ireland, Hungary, and Slovenia. The stakeholder panels will engage citizens and professionals in the co-design and testing of the WiseFood app, supporting the development of inclusive and feasible solutions for healthier and more sustainable food systems. In parallel, a household-level survey and expert focus groups will be conducted to explore user behaviors, preferences, and attitudes toward AI-driven dietary tools, and to gather expert insights on feasibility, acceptability, and equity considerations. These activities will directly inform the design and refinement of WiseFood digital prototypes.

#### Participants

Each LL site will recruit approximately 50 participants, drawn from 2 main groups:

1. Households (n=40 per site): Lay citizens from diverse household types, excluding individuals with professional nutrition backgrounds to minimize bias. Recruitment will ensure balanced representation across key household types: (1) single-person households, (2) single-parent families, (3) cohabiting couples without children, (4) cohabiting couples with children, and (5) households including individuals more than 65 years of age.
2. Expert group (n ≈ 10 per site): Professionals from sectors relevant to sustainable diets and digital health, such as nutrition, dietetics, public health policy, agriculture, and the food industry.

#### Procedures

A coordinated recruitment strategy will be implemented across study sites, with adaptations to suit local context. For the citizen cohort, recruitment will primarily be conducted online via targeted social media campaigns and digital posters, directing individuals to a designated project email address. Upon expressing interest, individuals will receive a confirmation email and further information about the project. In some cases, participants previously involved in LL studies may be approached directly to participate. Participant data will be securely managed using access-controlled spreadsheets, with site-specific access to ensure confidentiality. Following recruitment, participants will engage in 2 core activities:

1. Household survey: administered online, the survey includes 37 items across 6 domains: (1) demographics, (2) behavior and attitudes toward food choice, (3) attitudes toward AI in food choice, (4) preferred features and functionality of a digital food tool, (5) preferences for interaction when using a digital tool, and (6) barriers, preferences, and expectations for WiseFood (Multimedia Appendix 1).
  The survey was collaboratively developed across LL sites, reviewed by experts in nutrition and digital health for face and content validity, and translated by local teams to ensure linguistic and cultural appropriateness. Quantitative data will be analyzed descriptively, and open-ended responses will undergo content analysis.
2. Expert focus groups: conducted in person or via Microsoft Teams, sessions will last approximately 75 minutes and follow a semistructured topic guide covering: (1) professional experience of AI use in food, nutrition, and health promotion; (2) reactions to AI in the WiseFood context: trust, usefulness, and implementation at household level; (3) risks, barriers, and ethical considerations in the use of AI and WiseFood in food choice; (4) feasibility and enablers to the use of WiseFood; and (5) final reflections on the WiseFood tool (Multimedia Appendix 2). Participants will complete a brief demographics questionnaire prior to the session (Multimedia Appendix 3). Discussions will be audio-recorded, transcribed verbatim, and analyzed thematically using Braun and Clarke’s 6-step approach [29], with reflexivity and intercoder reliability maintained throughout.

### Phase 3: Co-Design Workshops and Iterative Feedback Loops

#### Study Overview

**Figure 3:**
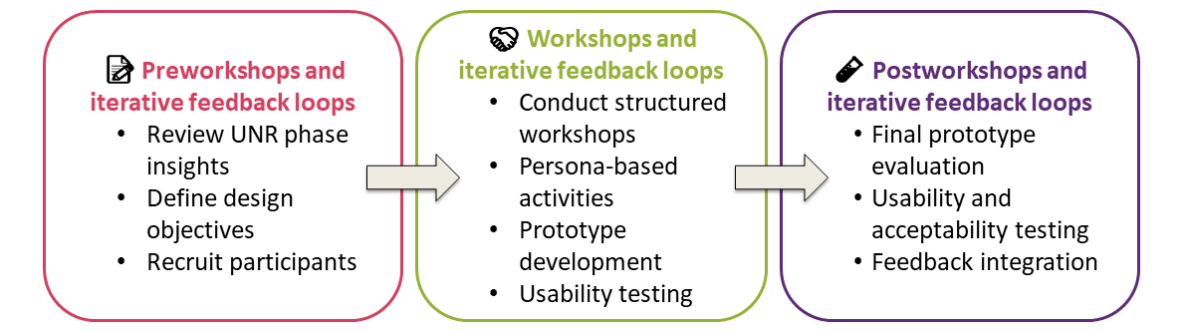
Co-design workshops and iterative feedback loops in the WiseFood project. UNR: User Needs and Requirement.

Phase 3 of the WiseFood project builds on established participatory design methodologies that focus on digital health, adapted from Bird et al [26] and Timon et al [30] (Figure 3). This phase aims to collaboratively generate, prototype, and iteratively refine the features and functionalities of the WiseFood digital platform, ensuring alignment with the real-world needs of users. Insights from the earlier UNR phase will guide the development of prototypes, which will be evaluated and refined through successive cycles of feedback and technical development. The co-design process will culminate in a functional prototype that will be evaluated for usability and acceptability in Phase 4.

#### Participants

Co-design participants will include representatives from previously engaged household members and expert stakeholders, while new stakeholders may also be recruited to ensure representation from diverse household types and socioeconomic backgrounds.

#### Procedures

A series of 1-day structured co-design workshops will be conducted by each LL site to generate ideas, concepts, and visualizations for the WiseFood platform. The overall process for planning and delivering the workshops is outlined in Table 2. Recruitment for co-design workshops will be guided by the principle of information power [31]. Preliminary analysis will be conducted after each session to determine whether participant contributions provide sufficient breadth and depth of insights to inform the co-design process. Workshops will conclude once additional sessions are unlikely to add meaningful refinement or novel directions for the intervention co-design process.

**Table 2:** Overview of the co-design workshop process across living lab sites.

Session title	Plan for delivery and facilitation	Session content
Introductions	Welcome participants, outline the workshop agenda, and explain the importance of participant contributions. 10-minute presentation to introduce the concept of co-design, its principles, and relevance to the WiseFood project. Researchers lead a discussion and Q&A^a^ to clarify research objectives and how participant input will shape the WiseFood platform.	Overview of the WiseFood project and workshop objectives. Introduction to co-design principles and the collaborative process. Emphasis on the value of participant perspectives for developing usable and effective tools.
Persona-based activities	Facilitated activities using the Generative Co-Design Framework [26]. Researchers guide participants through persona and scenario-based tasks to elicit needs, preferences, and suggestions. Fictional family personas are used to stimulate discussion and ensure diverse household types and behaviors are considered.	Ideation activities to generate ideas, concepts, and visualizations for the WiseFood platform. Stakeholders share insights into household food behaviors and FW^b^. Documentation of needs and requirements for user-centered design.
Iterative review and usability testing	Researchers present initial WiseFood prototypes. Participants provide feedback on usability, design, and relevance.	Hands-on review and testing of WiseFood prototypes. Collection of participant feedback for iterative refinement. Discussion of usability, effectiveness, and alignment with household needs.

^a^Q&A: question and answer.

^b^FW: food waste.

### Phase 4: Feasibility Study

#### Study Overview

**Figure 4:**
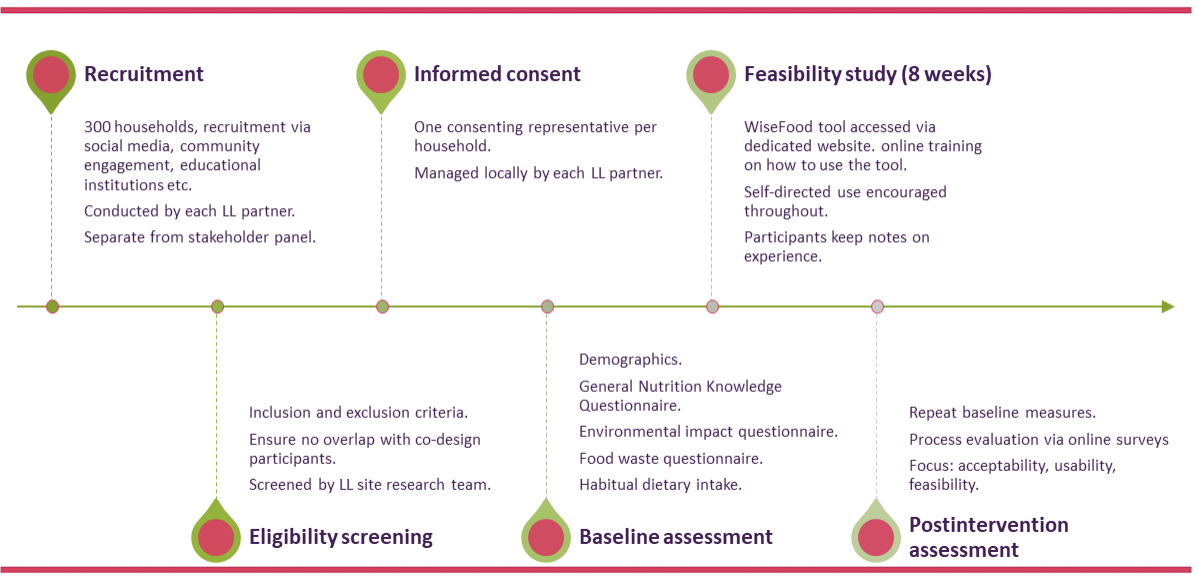
WiseFood feasibility study process. LL: Living Lab.

To assess whether the WiseFood app can be used as intended and is acceptable to users, feasibility is defined as the practicality of delivering the intervention and achieving engagement in real-world settings, aligning with WiseFood’s objective to support household-level food choices. To explore the feasibility, perceptions, and acceptability of WiseFood, a multicenter (RCSI, ESSRG, WF, and GP) 8-week single-arm cohort study will be conducted. The study design, population, recruitment procedures, baseline and follow-up assessments, intervention, and analysis plan are described in detail below. An overview of the feasibility study process is provided in Figure 4.

#### Population and Recruitment

A total of 300 households (n=100 per site) will be recruited. A recent scoping review by Chew et al (2023) [32] reported substantial variability in sample sizes across 17 interactive digital nutrition interventions (mean n=235). The proposed sample of 300 households is consistent with this range and, given the multisite design, supports stable estimation of feasibility outcomes overall and across LL sites. Eligible participants are adults aged 18 years or older who are the primary food decision-makers in their households and have internet access. Households will be excluded if any members follow medically prescribed diets (eg, diabetic, renal, gluten-free, high-protein and high-energy, and modified texture), or have dietary restrictions due to allergies, intolerances, vegan or vegetarian diets, or religious practices.

Recruitment will be conducted separately from the stakeholder panel involved in the co-design phase to avoid bias from prior exposure to the WiseFood app. As in other phases, an incentive will be offered to those who take part in Phase 4. Recruitment will follow procedures similar to those used for the stakeholder panel as described above, alongside targeted recruitment strategies to ensure representative participation across household types, age groups, ethnic backgrounds, socioeconomic status, and levels of digital literacy. One adult per household will complete the informed consent process.

#### Outcome Measures

Primary outcomes include feasibility metrics, recruitment, retention, and engagement, together with user acceptability measured through the Technology Acceptance Model (TAM) and qualitative feedback. Secondary outcomes include changes in nutrition knowledge (General Nutrition Knowledge Questionnaire; GNKQ), sustainability knowledge (Environmental Impact Questionnaire), dietary intake (Foodbook24 or national equivalents), and self-reported food waste practices. These indicators capture both the behavioral and perceptual dimensions of WiseFood use. The outcome measures, corresponding instruments, and assessment timepoints are summarized in Table 3.

**Table 3:** Evaluation and outcome measures for WiseFood feasibility study.

Measure			Details		Timepoint		
					Baseline (week 0)		Follow-up (Week 8)
**Demographics**							
	Demographic survey	Household type, age, gender, ethnic background, education level, and place of residence.		✓^a^			
**Primary outcomes**							
	Feasibility metrics	The primary feasibility endpoints are recruitment, retention, and engagement with the WiseFood tools. Recruitment will be defined as the proportion of eligible households enrolled out of those screened. Retention will refer to the proportion of enrolled households completing follow-up assessments at 8 weeks. Consistent with benchmarks reported in a similar digital health studies [33]. Engagement will be assessed using platform analytics, including frequency of use and interaction with core WiseFood features. The target is to recruit and retain 100 households per Living Lab (LL) site (n = 300 total). Acceptability will be measured quantitatively using the Technology Acceptance Model (TAM) and qualitatively with survey data [34].		✓		✓	
	Study evaluation	A process evaluation will be conducted through surveys where necessary to assess user experiences, perceived value, usability, acceptability, and gather suggestions for improvement.				✓	
**Secondary outcomes**							
	General Nutrition Knowledge Questionnaire (GNKQ) [35]	Pre- and postfeasibility study changes in nutrition knowledge will be assessed using the GNKQ.		✓		✓	
	16-item Environmental Impact Knowledge Questionnaire [36]	Pre- and postfeasibility study changes in sustainability knowledge will be assessed using a 16-item environmental impact questionnaire.		✓		✓	
	Dietary intake via Foodbook24 (or adapted national version) [37]	Changes in household dietary intake will be assessed using a Food Frequency Questionnaire (FFQ) delivered via the validated Foodbook24 platform.		✓		✓	
	Food Waste Questionnaire [38,39]	Household FW^a^ behaviors will be measured through self-reported surveys, exploring practices related to planning, purchasing, storage, and disposal, with the aim of determining whether use of the WiseFood applications contributes to a reduction in FW over the study period.		✓		✓	

^a^Check mark (✓) was used to differentiate whether a survey was required at a time point or not – an empty box indicated no, and a box with a check indicated yes.

^b^FW: food waste.

#### Baseline Assessments

At baseline (week 0), participants will complete a demographic survey, a GNKQ, a 16-item Environmental Impact Knowledge Questionnaire, and a habitual dietary intake record (Table 3). All instruments will be translated and culturally adapted by each LL partner to ensure relevance and accessibility.

#### Intervention

Participants will receive online training materials explaining how to access and use the WiseFood platform via a dedicated website. They will be instructed to interact with the platform as often as they find helpful over the 8-week period. Participants will be asked to keep notes on their experiences using WiseFood to support healthier and more sustainable food choices. A dedicated support email and phone line will be available throughout the study for technical assistance.

#### Follow-Up Assessments

At the end of the 8-week period, participants will be invited to repeat all baseline assessments and take part in a process evaluation through surveys to capture user experiences, perceived value, usability, acceptability, and suggestions for improvement (Figure 2; Table 3). To strengthen the validity and depth of our findings, data will be triangulated across methods, with qualitative data used to contextualize and interpret quantitative results, identifying shared patterns and differences.

#### Data and Statistical Analysis

All statistical analyses will be conducted using either R (Posit PBC) or Stata (StataCorp LLC), depending on institutional licensing availability. Primary outcomes will include feasibility (recruitment, retention, and engagement) and acceptability in recruited households. Recruitment will be defined as the proportion of eligible households enrolled among those screened. Retention will be defined as the proportion of households that complete the 8-week follow-up assessment. Retention rates will be interpreted in the context of comparable digital nutrition and health interventions [33]. Engagement will be assessed using platform analytics, including frequency of use and interaction with core WiseFood features. Acceptability will be assessed using TAM [34] survey scores and process evaluation surveys. Feasibility and acceptability outcomes will be summarized using proportions or means with 95% CIs.

Secondary outcomes (nutrition knowledge, sustainability knowledge, dietary intake, and FW behaviors) will be analyzed as change from baseline to 8 weeks in individual participants using regression models with appropriate adjustment. Estimated mean differences with 95% CIs will be reported.

Attrition and missing data will be documented overall and by LL site, with reasons for noncompletion summarized where available. Data management procedures will be harmonized across sites, and pooled analyses will adjust for the LL site.

## Results

The project is funded for a period of 3 years, from January 1, 2025, to December 31, 2027. It follows a 4-phase structure aimed at developing, refining, and testing a user-focused app through LL sites in Ireland, Hungary, and Slovenia.

Enrollment for Phase 1 of this study was completed in May 2025. A total of 50 individuals, including both members of the public and expert stakeholders, were recruited across each of the 3 LL sites to form diverse panels that represent a broad range of household types and relevant perspectives. Phase 2 ran from June to July 2025. During this phase, household participants completed an online survey, while the expert group attended one of a series of online focus groups. These results will be published separately at a later date.

Building on feedback from Phase 2, Phase 3 involves the co-design and iterative refinement of the app. This phase commenced in September 2025 and is expected to continue until June 2026.

Phase 4 is scheduled to begin in July 2026, with ethics approval to be submitted in advance at each participating site. New participants will be recruited across the LL sites to assess how the app performs in day-to-day use. This stage will test the practicality and effectiveness of the app, incorporating all refinements made throughout the previous phases. Phase 4 is expected to conclude by November 2027.

## Discussion

### Principal Anticipated Findings

This study describes the co-design and multicenter feasibility evaluation of the WiseFood app, developed to support healthier and more sustainable household food choices. The co-design phase is expected to produce a user-informed digital platform tailored to diverse household contexts. The feasibility phase is designed to assess recruitment, retention, engagement, and acceptability in real-world LL settings, and to generate preliminary estimates of change in nutrition knowledge, sustainability knowledge, dietary behaviors, and household FW practices over an 8-week period. The primary emphasis of this evaluation is to determine whether WiseFood can be implemented and engaged with as intended across different contexts.

### Comparison With Prior Work

Digital nutrition interventions have largely focused on individual behavior change, with comparatively limited attention to the collective and negotiated nature of household food decisions [14,15]. Few initiatives have integrated AI-supported personalization within a structured co-design framework across multiple real-world environments. By targeting households and embedding development and testing within LLs across three European countries, WiseFood seeks to address this gap. The combination of stakeholder engagement and AI-driven tailoring represents an innovative approach to supporting dietary decision-making within complex household settings.

### Strengths and Considerations

A key strength of this study is the integration of co-design with multicenter feasibility testing, enhancing contextual relevance and usability. The LL model supports evaluation under real-world conditions, while AI-supported personalization enables tailored guidance responsive to household needs.

As the primary focus is engagement and acceptability, the feasibility design prioritizes assessment of recruitment, retention, user interaction, and perceived usefulness rather than formal effectiveness testing. The 8-week duration allows examination of short-term engagement patterns, although longer-term use and sustained behavioral change are beyond the scope of this phase. Variability in digital literacy and household routines may influence engagement; however, iterative stakeholder involvement and a flexible interaction model are intended to support accessibility across diverse user groups.

### Future Directions

The findings from this feasibility study will inform refinement of the WiseFood platform and optimization of implementation strategies across LL contexts. Further evaluation will be required to examine longer-term engagement patterns and behavioral outcomes, including exploration of how AI-supported dietary advice may contribute to household-level decision-making in relation to common nutrition-related conditions.

### Conclusions

This study outlines the co-design and multicenter feasibility evaluation of WiseFood, an AI-supported digital platform targeting household-level food decision-making. By embedding development within LLs and incorporating stakeholder input throughout, the project prioritizes contextual relevance, usability, and real-world applicability. The feasibility phase will assess engagement and acceptability to determine whether the platform functions as intended across diverse household settings. Through the integration of co-design and AI-driven personalization, WiseFood offers a novel approach to supporting healthier and more sustainable food choices at the household level.

## Data Availability

No datasets are available at this stage. Due to privacy restrictions, individual-level data will not be publicly shared; aggregated data may be provided upon request.

## Appendix

Multimedia Appendix 1 WiseFood User Needs and Requirements Survey. Household level.

Multimedia Appendix 2 WiseFood Stakeholder Focus Group Topic Guide.

Multimedia Appendix 3 WiseFood Stakeholder Focus Group Demographic Questions.

## Abbreviations

AI: artificial intelligence
ESSRG: Environmental Social Science Research Group
FFQ: Food Frequency Questionnaire
FW: food waste
GE: green eating
LL: Living Lab
RCSI: Royal College of Surgeons in Ireland
TAM: Technology Acceptance Model
UNR: User Needs and Requirements
WF: Wasteless Foundation
