# Supplementary material for: Evaluation of an AI-Supported Nutrition Application (WiseFood) in a Living Lab Context: Protocol for a User Needs Assessment, Co-Design, and Feasibility Testing
Source: JMIR Res Protoc. 2026 Apr 27;15:e88810. doi: 10.2196/88810 (PMC13161834; doi:10.2196/88810)
Supplement: Multimedia Appendix 1 [file resprot_v15i1e88810_app1.docx]

**WiseFood User Needs and Requirements Survey – Household Level**

**Section 1: Demographic Information**

1. **What country do you live in?**

- Ireland
- Hungary
- Slovenia

1. **Age:**

- 18 – 24 years
- 25 – 34 years
- 35 – 49 years
- 50 – 64 years
- 65 years or older

1. **Gender:**

- Male
- Female
- Other: _______
- Prefer not to say

1. **What is your ethnic background?** *This question is asked only to help us describe the overall diversity of people who respond to the survey. Please select the option that best describes your ethnic or cultural background:*

White

- Irish
- Irish Traveller
- Roma
- Any other White background *(Please specify*) ___________________________________

Black or Black Irish:

- African
- Any other Black background *(Please specify*) ___________________________________

Asian or Asian Irish:

- Chinese
- Indian
- Pakistani
- Bangladeshi
- Any other Asian background *(Please specify*) ___________________________________

Other, including mixed group/background:

- Arabic
- Mixed *(Please specify*) ___________________________________
- Other *(Please specify*) ___________________________________
- Prefer not to say

1. **What type of household do you represent?**

- Single person household (includes person living alone or person living in shared household). *A* ***shared household*** *refers to a living arrangement where a* ***single person resides with others*** *(who are not part of their family or household unit) e.g. house share)*
- Cohabiting couple household (no children in household)
- Single parent household
- Cohabiting couple with children household
- Older adult/s household (65 years or older)
- Other household type – please describe ________________________________________

1. **Highest level of education completed:**

- No formal education/training
- Primary
- Lower secondary (Group; Inter; Junior Certificate)
- Upper secondary (Leaving Certificate)
- Technical or vocational
- Advanced certificate/completed apprenticeship
- Higher certificate
- Ordinary Bachelor Degree or National Diploma
- Honours Bachelor Degree or Professional Qualification or both
- Postgraduate Diploma or Master’s Degree
- Doctorate (Ph.D.) or Higher

1. **Which of the following best describes the area where you live?"**
   (Please select one option)

- A city
- A large town (population over 10,000)
- A small town (population under 10,000)
- A village
- A rural area / countryside

1. **How health conscious do you consider yourself to be?**

- Not health conscious at all
- Slightly health conscious
- Moderately health conscious
- Very health conscious
- Extremely health conscious

1. **Do you follow any specific dietary practices? (e.g., vegetarian, vegan, gluten-free, etc.):**

- Yes
- No

***If yes, please select which practice(s)***

- Vegetarian
- Vegan
- Gluten-free
- Dairy-free
- Other (Please specify)______________________________________________

1. **Do you have any health conditions that influence your diet? (e.g., diabetes, hypertension, allergies, sensitivities, etc.)**

- Yes
- No

***If yes, please select condition(s)***

- Diabetes
- Hypertension
- Coeliac Disease
- Allergies
- Sensitivities
- Other (Please specify)______________________________________________

1. **How comfortable are you using the following types of technology?** Please rate your comfort level with each activity, where 1 = Very uncomfortable, 2 = Uncomfortable, 3 = Neutral, 4 = Comfortable, 5 = Very comfortable.

| *Activity* | *1* | *2* | *3* | *4* | *5* |
| --- | --- | --- | --- | --- | --- |
| Using websites like Google |  |  |  |  |  |
| Using social media platforms like Facebook or TikTok |  |  |  |  |  |
| Using personal digital devices related to health (e.g. smartwatches, step counters, etc.) |  |  |  |  |  |
| Using personal health apps on your mobile phone (e.g. sleep tracking, calorie monitoring, etc.) |  |  |  |  |  |
| Interacting with online services (e.g. online banking, renewing a passport etc.) |  |  |  |  |  |

**Section 2: Behaviour & Attitudes**

1. **Please rate how important each of the following factors is to you when making food choices.** 1 = Not important at all, 2 = Slightly important, 3 = Moderately important, 4 = Very important, 5 = Extremely important

| *Factor* | *1* | *2* | *3* | *4* | *5* |
| --- | --- | --- | --- | --- | --- |
| Health (e.g. nutritional value, balanced diet) |  |  |  |  |  |
| Price (e.g. affordability, budget-friendliness) |  |  |  |  |  |
| Convenience (e.g. quick to prepare, easy to access) |  |  |  |  |  |
| Sustainability (e.g. low carbon footprint, eco-friendly packaging) |  |  |  |  |  |
| Taste & Sensory Appeal (e.g. flavour, texture, aroma) |  |  |  |  |  |
| Natural Ingredients (e.g. no additives or preservatives) |  |  |  |  |  |
| Mood Impact (e.g. comfort food, stress eating) |  |  |  |  |  |
| Origin of product (e.g. locally sourced) |  |  |  |  |  |

1. **Have you ever used digital tools (e.g., apps or websites) to help with food choice?**

- Yes
- No

***If yes, which ones did you enjoy?***

________________________________________________________________________

***If no, would you be interested in using digital tools (e.g., apps or websites) to help with food decisions?***

- Yes
- No

1. **How often do you prepare meals at home in a typical week?**

- Never
- 1–2 times
- 3–5 times
- More than 5 times



**Section 3: Attitudes on Artificial Intelligence in Food Choice Decision Making**

1. **Do you currently use Generative AI (e.g. ChatGPT, Copilot etc.)?**

- Yes
- No

1. **Do you currently use Generative AI (e.g. ChatGPT, Copilot etc.) to help with food choice?**

- Yes
- No

1. **If you answered yes to Q16, what was your experience of this? (Single choice)**

- Very reliable
- Somewhat reliable
- Neutral/mixed
- Unreliable
- Irrelevant to my needs
- Other (*please specify) _*_________________________________

1. **How comfortable would you feel using an AI tool to guide your food choices? (Single choice)**

- Very uncomfortable
- Somewhat uncomfortable
- Neutral
- Somewhat comfortable
- Very comfortable

1. **What concerns, if any, would you have about using AI to guide your food choice? (Select all that apply)**

- Trust
- Privacy
- Accuracy
- Relevance
- Other *(please specify) _______________________________________________*

1. **What would increase your trust in AI-generated food advice? (Select all that apply)**

- Expert input
- Transparency
- Personalisation
- User reviews
- Government/health authority approval
- Content known to be accurate based on scientific research and guidelines
- Nothing – I would not trust AI-generated food advice
- Other *(please specify) _______________________________________________*

1. **Where do you typically get your information about nutrition and food? (Select all that apply)**

- Friends or family
- Healthcare professionals (e.g., doctor, dietitian)
- Social media (e.g., Instagram, TikTok, YouTube)
- Online articles or websites
- TV or radio
- Podcasts
- Academic or scientific publications
- I don’t seek out nutrition or food advice
- Other (*please specify*): ____________

1. **Which sources would you prefer an AI tool to use when providing nutrition information? (Select all that apply)**

- Scientific research or peer-reviewed studies
- Government or public health guidelines (e.g., HSE, SafeFood, FSAI, WHO)
- Academic institutions or universities
- Food product labels or manufacturer data
- I’m not sure
- Other (please specify): ____________

1. **Do you usually read the labels on food products before buying or eating them?**

- Yes
- No

***If yes, what do you look for?***

- Ingredients
- Nutrition information (e.g. calories, fat content, protein)
- Traffic light system
- Country of origin
- Fairtrade
- Other (*please specify*)____________________________________________________



**Section 4: Features & Functionality**

1. **Which of the following features would be most useful to you in supporting food choice at a household level?** (Select all that apply)

- Meal suggestions / Recipe ideas
- Weekly meal plans
- Recipes adapted to be more environmentally friendly
- Recipes tailored to my dietary preferences
- Recipe adjusted to fit my budget
- Shopping lists
- Food waste reduction tips
- Search engine for nutrition queries
- Nutrition composition of meals
- Scores showing how sustainable recipes are

1. **Who is primarily responsible for making food decisions in your household?** (Select one)

- I make most of the food decisions myself
- Food decisions are made jointly with all household members
- Food decisions are made together with some household members

***If you share food decisions with other members of your household, with whom in your household do you usually make food choices?***

*________________________________________________________________________*

1. **Would you prefer the digital tool (e.g., app or website) to be used by an individual on behalf of the household or multiple users**? (Single choice)

- Individual user
- Multiple users
- Other – please explain:________________ ________________________________________



**Section 5: Preferences for Interaction**

1. **Would you prefer interacting with the tool via** (Select all that apply)

*(images and descriptions of each to be provided to guide the respondent in answering these questions)*

- A chat-based (e.g., text conversation) interface
- A visual dashboard (visual layout with icons and data)
- Simple notifications/reminders
- A combination of all of these
- Other ____________________________________________________________

*(WiseFood applications images with descriptions presented to the respondent before answering this question – could this be a short video with voiceover?)*

1. **How often would you realistically use a tool like this in your daily or weekly routine? (**Single choice)

- Daily
- Weekly
- A few times per month
- Monthly
- Rarely
- Never *(If never, why?) ____________________________________________________*

1. **If these tools were accessible via a web application, in what way do you think you would access these tools?** *(Select all that apply)*

- Smartphone
- Tablet
- Laptop
- Virtual assistant – google home etc.
- Other (please specify) __________________________________________________

1. **If there are children in your home, would you like them to interact with the tools?**

- Yes
- No
- Not applicable

***If no, please explain why not:*** ______________________________________________



**Section 6: Barriers, Preferences, and Expectations for WiseFood**

1. **What features would make a digital tool most useful or relevant to your daily food choices?** *(Select all that apply)*

- Personalised Nutrition Tracking
- Meal Planning & Suggestions
- Shopping List Generation
- Sustainability Insights
- AI Recipe Generator
- Reminders & Notifications
- Other *(please specify) _____________________________________________*

1. **Is there anything that would prevent you from using a tool like WiseFood?**

- Yes
- No

1. **If yes, what would prevent you from using a tool like WiseFood?** (Select all that apply)

- Lack of trust in AI
- Privacy concerns
- Lack of time
- App fatigue
- Complexity or Usability
- Lack of interest
- Other *(please specify) _____________________________________________*

1. **Do you have any suggestions or expectations for how this type of tool should support you or your household?** (Select all that apply)

- Ability to manage preferences of all household members
- Budget and waste conscious features
- Realistic meal planning
- Family engagement tools e.g. challenges for the week
- Offline options
- Other *(please specify) _____________________________________________*

1. **In terms of training on how to use these tools, what format would be most useful to you**? (Select all that apply)

- Short pre-recorded video tutorials (2-5 mins each)
- Step-by-step guide the first time you open the app
- In-App tips and pop-ups
- Answers to frequently asked questions and searchable help centre
- Downloadable PDF
- Live online video tutorials with a member of staff
- Other *(please specify) _____________________________________________*

1. **If you had access to a tool capable of answering questions about nutrition, what are three or more questions you would be interested in asking?** *(optional)*

_______________________________________________________________________

1. **Is there anything else you would like to mention related to this topic?**

____________________________________________________________________
