# Supplementary material for: Evaluation of an AI-Supported Nutrition Application (WiseFood) in a Living Lab Context: Protocol for a User Needs Assessment, Co-Design, and Feasibility Testing
Source: JMIR Res Protoc. 2026 Apr 27;15:e88810. doi: 10.2196/88810 (PMC13161834; doi:10.2196/88810)
Supplement: Multimedia Appendix 2 [file resprot_v15i1e88810_app2.docx]

**WiseFood Stakeholder Focus Group Topic Guide**

**Target participants:** Nutritionists, dietitians, public health professionals, policymakers, food industry representatives

**Purpose**: To explore expert views on the development, implementation, and acceptability of AI tools supporting healthy and sustainable food choices at the household level.

**Time:** 60 – 90 mins (allowing for 10–15-minute discussion per section)

**Location:** Focus groups hosted via Zoom/Teams or in person

**Participants:** Optimum is 4 – 6 participants per focus group

-------------------------------------------------------------

**Introduction:**

**Introduce researchers and purpose of interview:**

Welcome and thank you for participating in this focus group. We are [researcher names]. This study is part of the WiseFood project, which aims to develop and test a digital platform powered by artificial intelligence (AI) to support healthier and more sustainable food choices at the household level. The information gathered will directly inform the development of the WiseFood applications, helping to ensure they are evidence-based, user-friendly, and practically useful.

**Informed consent:**

Our focus group discussion will take approximately 90 minutes today and will be audio-recorded and transcribed. You may skip any questions or topics you do not wish to discuss and may leave the session at any time. Everything we discuss today will remain confidential, and we will endeavour to remove any names or other potentially identifying information from the focus group transcript. Only summarised information will be provided in any reports resulting from this project.

**Clarify that all participants are happy to proceed and begin recording/transcription**

---------------------------------------------------------------

**Key terms:**

**Household:** A household is a group of people who live together in the same home and share living arrangements, such as meals, bills, and responsibilities. It can also be just one person living alone.

**Single-person household**: One person living alone, or within a shared household of unrelated people who share expenses.

**Single-parent household**: One parent living with one or more children.

**Cohabiting couple – no children household**: A couple living together without children.

**AI:** Artificial Intelligence is when computers or machines are made to think and learn like people so they can help us do things—like talk, play games, or find answers. Chatbots that answer customer service questions, virtual assistants like ChatGPT or Co-Pilot, and voice assistants like Siri or Alexa are all examples of AI.

---------------------------------------------------------------

| **Questions** | **Prompts and/or additional questions (optional)** |
| --- | --- |
| 1. **Professional Experience and AI Use -**  - Can you briefly describe your role and experience in relation to food, nutrition, or health promotion? - When you are looking for information to support your work—whether it's for making decisions, giving advice, or staying up to date—what sources do you use? - From your perspective, what are some of the strengths and limitations (or gaps) in current approaches to promoting healthy eating? - What kinds of AI tools do you use in your work, or outside of work (if any)? What role do they play (if any)? | What does “healthy eating” mean to different groups you work with?  What helps people maintain healthy eating habits over time?  In your experience, what motivates people to change their eating habits?  Have you seen or heard of members of the general public using AI tools (e.g., ChatGPT, food apps) to support food choices? What are your impressions? |
| 1. **Reactions to AI in the WiseFood Context**   *(Brief presentation on WiseFood project and apps)*   - What are your initial thoughts on using AI to support healthy and sustainable food choices at the household level? - WiseFood looks up trusted nutrition sources and combines them with AI to give tailored advice—kind of like an expert assistant that checks its facts before answering. How does that influence your trust in what it tells users? - What are the pros / cons to having tools like this in households or the workplace? - We are considering testing this tool in the household. What are your thoughts on this? | What comes to mind when you hear “AI supporting healthy eating”?  What information would help you to determine if a recommendation is trustworthy?  How important is it for users to *understand how* a recommendation is generated?  What problems do current tools fail to solve for you or your community? How might WiseFood differ from existing tools? |
| 1. **Risks, Barriers and Ethical Considerations**  - What concerns do you have about using AI in this context? - Do you foresee any barriers to adoption or acceptability for the general public? - In your opinion, who do you think would benefit the most from tools like WiseFood, and who might be left out? | *Prompt if needed:* accuracy, trust, data privacy, misinformation, over-reliance on technology  *Prompt if needed:* consider culture, language, finance, digital access |
| 1. **Feasibility and Enablers, for Adoption**  - What features or functions do you think would make this kind of tool useful and accessible? - How could this type of tool be integrated into national health promotion efforts? - How could the tool support *long-term engagement*? | Prompt: What about nutrition labeling systems (e.g., Nutri-Score, Multiple Traffic Light)?  What about similar scores related to sustainability (e.g., Eco-Score, Carbon Labels)? |
| **5. Final Reflections**   - What do you think success would look like for a tool like WiseFood? - Are there any examples or lessons from your own work or sector that might inform the development or use of WiseFood? - What is the top question you would like to ask if you had access to a Question Answering tool for nutrition? - Is there anything else you’d like to share on this topic? | How would you measure whether it’s working well?  What would make someone *keep using* the tool long-term?  What’s the biggest mistake the WiseFood developers should avoid?  If you could have one tool to support food-related decisions, what would it help with? |

**Close:**

Today we wanted to talk to you about how people and key stakeholders perceive food decision-making, and how digital tools might support this process. Is there anything else you would like to discuss? Do you have any questions for us?

[Researcher to outline next steps].

That brings our focus group to a close. Thank you for your time and your contributions today.
