# Supplementary material for: Evaluation of an AI-Supported Nutrition Application (WiseFood) in a Living Lab Context: Protocol for a User Needs Assessment, Co-Design, and Feasibility Testing
Source: JMIR Res Protoc. 2026 Apr 27;15:e88810. doi: 10.2196/88810 (PMC13161834; doi:10.2196/88810)
Supplement: Multimedia Appendix 3 [file resprot_v15i1e88810_app3.docx]

**Stakeholder Focus Group – Demographics**

[to be sent to attendees with consent form ahead of focus group and completed in advance]

**In which country do you currently work?**
(Dropdown)

**What is your age?**

- Under 25
- 25–34
- 35–44
- 45–54
- 55–64
- 65 or older

**What is your gender?**

- Male
- Female
- Non-binary / Third gender
- Prefer not to say
- Prefer to self-describe: ______________________

**Professional Background**

What is your current job title or role?
(Open-ended)

**How many years of experience do you have in the food/nutrition field?**

- Less than 1 year
- 1–5 years
- 5-10 years
- More than 10 years

**Which of the following best describes your primary area of work?**

- Public Health Nutrition
- Clinical Nutrition/Dietetics
- Research/Academia
- Food Science
- Technology
- Policy/Regulation
- Other (please specify): ______________________

**What type of organisation do you currently work for?**

- Hospital/Healthcare Facility
- Government Agency
- University/Research Institution
- Food/Nutrition Industry
- Technology
- Non-profit/NGO
- Other (please specify): ______________________
